# Effect of heat and moisture exchanger (HME) positioning on inspiratory gas humidification

**DOI:** 10.1186/1471-2466-6-19

**Published:** 2006-08-08

**Authors:** Daisuke Inui, Jun Oto, Masaji Nishimura

**Affiliations:** 1 Department of Emergency and Critical Care Medicine, The University of Tokushima Graduate School, 3-18-15 Kuramoto, Tokushima, 770-8503, Japan

## Abstract

**Background:**

In mechanically ventilated patients, we investigated how positioning the heat and moisture exchanger (HME) at different places on the ventilator circuit affected inspiratory gas humidification.

**Methods:**

Absolute humidity (AH) and temperature (TEMP) at the proximal end of endotracheal tube (ETT) were measured in ten mechanically ventilated patients. The HME was connected either directly proximal to the ETT (Site 1) or at before the circuit Y-piece (Site 2: distance from proximal end of ETT and Site 2 was about 19 cm) (Figure. 1). Two devices, Hygrobac S (Mallinckrodt Dar, Mirandola, Italy) and Thermovent HEPA (Smiths Medical International Ltd., Kent, UK) were tested. AH and TEMP were measured with a hygrometer (Moiscope, MERA Co., Ltd., Tokyo, Japan).

**Results:**

Hygrobac S provided significantly higher AH and TEMP at both sites than Thermovent HEPA. Both Hygrobac S and with Thermovent HEPA provided significantly higher AH and TEMP when placed proximally to the ETT.

**Conclusion:**

Although placement proximal to the ETT improved both AH and TEMP in both HMEs tested, one HME performed better in the distal position than the other HME in the proximal position. We conclude the both the type and placement of HME can make a significant difference in maintaining AH and TEMP during adult ventilation.

## Background

During normal breathing, the upper airway effectively deliver inspired air to the lower respiratory tract (at the carina) condition to approximately 32°C with a relative humidity (RH) of more than 90% (absolute humidity (AH) 30.4 mg/L)[1]. As this inspired air enters the alveoli, it is warmed to body temperature (about 37°C) and reaches 100% humidification [2,3]. When the upper airways are circumvented, delivery of dry gas to the lungs has been associated with damage to the tracheobronchial mucosa [4-7]. Consequently, when the natural humidification of the upper airways is bypassed by an endotracheal or tracheostomy tube, artificial humidification of inspiratory gas is essential for mechanically ventilated patients. As an alternative to heated humidifiers, heat and moisture exchangers (HME) have been widely adopted in intensive care units (ICU) [8]. HME performance is influenced by many factors, including model and brand, duration of service, body temperature, tidal volume (V_T_), minute volume (MV), and room temperature. For most mechanically ventilated patients, however, HMEs are considered to perform well in warming and humidifying inspired air. Intuitively, we considered that placing the HME close to the endotracheal tube (ETT) would better humidify and warm ingoing gas than distal placement on the ventilator circuit. When we looked for evidence to confirm our intuition, however, we found few reports of the effects of HME positioning on the quality of inspired gas. This led us to concretely investigate the effects of HME positioning on inspiratory gas humidification in mechanically ventilated patients.

## Methods

Ten mechanically ventilated adults were enrolled in this study. All patients received mechanical ventilation at least for 24 hours before the tests were carried out. In our unit, we usually use HME for all mechanically ventilated adult patients and the procedures did not require any special medical intervention, so no Institutional review board (IRB) approval of the protocol was necessary. We did, however, secure informed consent from the next of kin of all participants.

**Figure 1:**
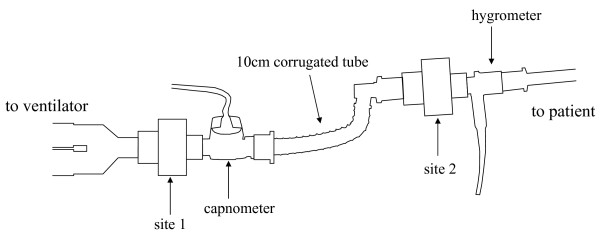
A heat and moisture exchanger (HME) was connected directly to the ETT (Site 1) and to the intersection of the Y piece and a 10 cm length of corrugated tube (Site 2).

We tested two HMEs, Hygrobac S (Mallinckrodt Dar, Mirandola, Italy) and Thermovent HEPA (Smiths Medical International Ltd., Kent, UK), in two locations on the ventilator circuit. Each HME was connected either directly proximal to the ETT (Site 1) or after 10 cm of flexible tube at before the circuit Y-piece (Site 2: the distance between the proximal end of the ETT and Site 2 was about 19 cm) (Figure. 1). The two HMEs and site of HME were randomly assigned to different patients.

We measured temperature (TEMP) and RH with a rapid response capacitance-type moisture sensor (Moiscope, MERA Co., Ltd., Tokyo, Japan), placed at the proximal end of the ETT (Figure. 1). The time constant of the hygrometer was 0.6 sec when humidity decreased, and 0.76 sec when humidity increased. After readings on the screen of the hygrometer for AH and TEMP of inspired gas stabilized, we waited 10 more minutes, and AH and TEMP readings were recorded. Inspiratory flow was also recorded via signal output of the ventilators, and it was used to recognize inspiratory phase. Values for ten consecutive breaths were recorded, and AH and TEMP results were averaged.

We did not include a flow-meter in the ventilator circuit, but recorded the V_T_, and MV values as shown on the ventilator display. All patients were mechanically ventilated by either a Servo 300 or a Puritan-Bennett 7200 ae. Room temperature was monitored by another hygrometer of same brand, and body temperature was measured by an ET-C202P thermometer (TERUMO Co., Ltd., Tokyo, Japan).

Values are expressed as means ± standard deviation (SD). Statistical analysis was applied with one-way ANOVA and p < 0.05 was considered significant.

## Results

**Figure 2:**
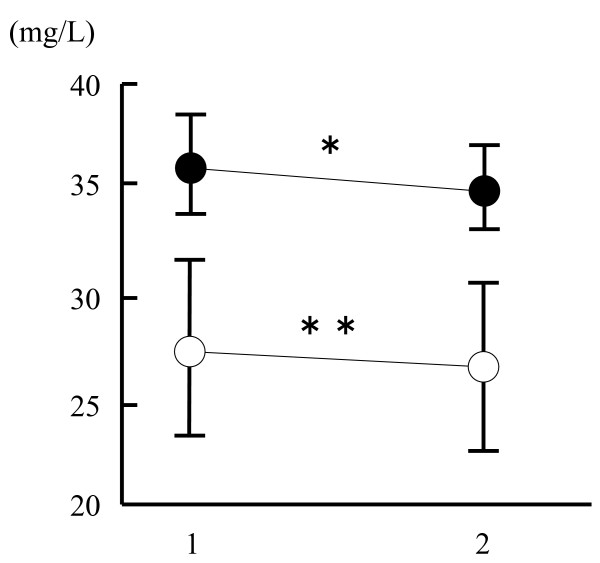
Absolute humidity (AH) with Hygrobac S or Thermovent HEPA placed at Site 1 or Site 2. AH was significantly higher when Hygrobac S (closed circle)(*p < 0.05) was placed at Site 1 than at Site 2, and when Thermovent HEPA (open circle)(**p < 0.05) was placed at Site 1 than at Site 2. Patient N = 10. Data presented as mean ± SD.

Table 1 shows the basic characteristics of the patients. AH values for Hygrobac S were 36.5 ± 1.96 mg/L when positioned at Site 1, and 35.1 ± 1.56 mg/L at Site 2 (p < 0.05)(Figure. 2). With Thermovent HEPA, AH values were 27.7 ± 4.23 mg/L for Site 1 and 26.8 ± 4.41 mg/L for Site 2 (p < 0.05)(Figure. 2). Positioned at Site 1, use of Hygrobac S gave significantly higher AH values than Thermovent HEPA.

**Figure 3:**
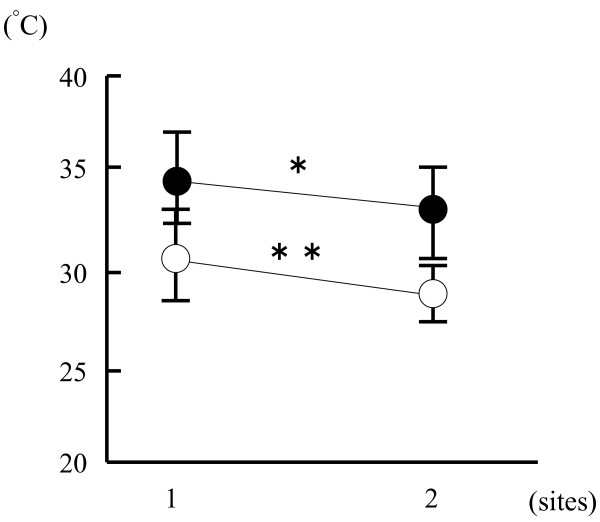
Temperature (TEMP) with Hygrobac S or Thermovent HEPA placed at Site 1 or Site 2. TEMP was significantly higher when Hygrobac S (closed circle)(*p < 0.05) was placed at Site 1 than at Site 2, and when Thermovent HEPA (open circle) was (**p < 0.05) was placed at Site 1 than at Site 2. Patient N = 10. Data presented as mean ± SD.

TEMP values were 34.1 ± 1.74°C with Hygrobac S positioned at Site 1, and 33.1 ± 1.86°C at Site 2, (p < 0.05)(Figure. 3). With Thermovent HEPA, similar values were 31.3 ± 1.85°C for Site 1 and 29.0 ± 0.85°C for Site 2 (p < 0.05) (Figure. 3). Positioned at Site 1, use of Hygrobac S gave significantly higher TEMP values than Thermovent HEPA.

For each patient, during the measurement protocol, whatever the HME type and positioning, no significant differences in body temperature, room temperature, V_T_, and MV were found.

## Discussion

When either HME was used at Site 1, AH and TEMP were significantly higher than when location was at Site 2. This finding supports our intuitive assumption.

Although we assumed that AH and TEMP are higher when an HME is placed directly at the proximal end of the ETT, because tracheal secretions may easily contaminate the HME and even block it, we do not always place the HME here for mechanically ventilated patients except in operation room (OR). The closed-suction system that we use actually makes it impossible to connect the HME directly to the ETT. In practice, this connection is achieved with a corrugated tube (of about 10 cm), placed between the HME and the Y-piece.

While we do not know how much vapor is lost at each HME site, with either of the tested HMEs, we found that 10 cm of tube decreased AH by 1–2 mg/L and TEMP by 1°–2°C. This amount of lost is relatively small. Although we did not measure the temperature drop across the corrugated tube, 2–3°C might fall. As the temperature decreased, RH increased and preserved AH.

Various guidelines exist for humidification during mechanical ventilation. For example, ANSI (American National Standards Institute) recommends AH values of ≥30 mg/L; AARC (American Association for Respiratory Care) recommends AH values of ≥30 mg/L and inspiratory temperature at ≥30 ± 2°C; while (ISO: International Standards Organization) prefers AH values at ≥33 mg/L. Two systems, heated humidifier (HH) and HME are available for warming and humidifying gases delivered to mechanically ventilated patients. Even though thick secretions from patients are likely to occlude the tubes of HMEs, Kirton et al. have reported that the use of HMEs is more cost-effective than using HHs [9]. Meanwhile, the incidence of ETT occlusion when HMEs are used is lower than when HHs are used [10]. The performance of HMEs has been generally improving and this may partially account for the lower rate of ETT occlusion with HMEs. In the present study, Hygrobac S supplied 36.5 ± 1.96 mg/L of vapor when positioned at Site 1 and 35.1 ± 1.56 mg/L at Site 2: these values are higher than the values recommended in the previously mentioned guidelines. In the case of airleak, however, passive humidifier like HME cannot maintain adequate humidification, and HH should be chosen.

Hygrobac S met the guidelines even at Site 2, and so can facilitate the use of a closed-suction system. Thermovent HEPA was unable to match the guidelines even when connected directly to the ETT. In our hospital, the Hygrobac S was adopted by the ICU and OR, and the Thermovent HEPA only by the OR. While the Hygrobac S uses glass fiber, Thermovent HEPA uses glass. Lemmens et al. have reported significant differences in performance for the different HMEs[7]. It is possible that while some HMEs supply sufficient vapor saturation when located close to the ETT, they are unable to satisfy the guidelines when located more distantly.

Ünal et al. have reported that humidification efficiency of HMEs decreases as V_T _and MV increase [8]. In the present study, the underlying diseases differed from those in the Ünal report and while, rather than contending with significant variations in V_T _and MV, we maintained constant V_T _and MV during the measurements. Consequently, in this study, we are unable to report on the effect of V_T _and MV on humidification.

## Conclusion

Based on our findings, HMEs appear to be more effective when placed proximal to the ETT. When we use a distal position to accommodate closed suction catheters, we must select an HME that works well in this position.

## Competing interests

The author(s) declare that they have no competing interests.

## Authors' contributions

DI was responsible for the experimental conception and design, analyzed and interpreted the data, and worked up the manuscript.

JO contributed to the design and analyzed and interpreted the data.

MN was responsible for the clinical assessment of patients, carried out critical revision of the study for important intellectual content and was responsible for the acquisition of data.

## Abbreviations

RH: reralive humidity

AH: absolute humidity

HME: heat and moisture exchanger

ICU: intensive care unit

V_T_: tidal volume

MV: minute volume

ETT: endotracheal tube

IRB: Institutional review board

TEMP: temperature

SD: standard deviation

OR: operation room

ANSI: American National Standards Institute

AARC: American Association for Respiratory Care

ISO: International Standards Organization

HH: heated humidifier

## Pre-publication history

The pre-publication history for this paper can be accessed here:



## Figures and Tables

**Table 1:** Patient profile

No.	Diagnosis	Gender	Age (y)	BW (kg)	BT(°C)	RT(°C)	Vt(mL)	MV (L)
1	cavernous angiomapo.	M	32	60	36.8	26.2	600	8.1
2	acute bronchitis	F	26	29	36.0	27.0	230	2.8
3	hypertrophic CM	M	12	70	38.0	29.1	450	8.1
4	gastric cancer po.	M	74	75	37.4	29.7	530	9.1
5	PE po.	M	55	55	36.3	26.1	700	11.3
6	AAA po.	M	56	62	35.0	26.0	550	7.0
7	IA po.	M	72	72	37.2	26.0	520	7.8
8	pneumonia	M	68	55	36.6	27.0	260	8.7
9	meningitis	M	74	51	36.8	26.0	553	10.3
10	AR po.	M	42	61	36.5	26.2	380	9.0
Mean			51.1	59.0	36.7	26.9	477.3	8.2

po. postoperation, CM: cardiomyopathy, PE: pulmonary emobolism, AAA: abdominal aortic aneurysm, IA: intracranial aneurysm, AR: aortic regurgitation, M: male, F:female, BW: body weight, BT: body temperature, RT: room temperature, Vt: tidal volume, MV: minute volume
